# Hydroxypropyl-Beta-Cyclodextrin Reduces Inflammatory Signaling from Monocytes: Possible Implications for Suppression of HIV Chronic Immune Activation

**DOI:** 10.1128/mSphere.00497-18

**Published:** 2018-11-07

**Authors:** Flávio Lemos Matassoli, Ihid Carneiro Leão, Bruno Braz Bezerra, Richard B. Pollard, Dieter Lütjohann, James E. K. Hildreth, Luciana Barros de Arruda

**Affiliations:** a Departamento de Virologia, Instituto de Microbiologia Paulo de Góes, Universidade Federal do Rio de Janeiro (UFRJ), Rio de Janeiro, Rio de Janeiro, Brazil; b Department of Molecular and Cellular Biology, University of California, Davis, Davis, California, USA; c Department of Internal Medicine, University of California, Davis School of Medicine, Sacramento, California, USA; d Institute for Clinical Chemistry and Clinical Pharmacology, University Clinics of Bonn, Bonn, Germany; e Department of Internal Medicine, Meharry Medical College, Nashville, Tennessee, USA; Beth Israel Deaconess Medical Center

**Keywords:** HIV, beta-cyclodextrin, monocytes, inflammation, human immunodeficiency virus

## Abstract

Monocytes from HIV-infected patients produce increased levels of inflammatory cytokines, which are associated with chronic immune activation and AIDS progression. Chronic immune activation is often not restored even in patients showing viral suppression under ART. Therefore, new therapeutic strategies to control inflammation and modulate immune activation are required. Hydroxypropyl-beta-cyclodextrin (HP-BCD) is a cholesterol-sequestering agent that has been reported to be safe for human use in numerous pharmaceutical applications and that has been shown to inactivate HIV *in vitro* and to control SIV infection *in vivo*. Since cellular cholesterol content or metabolism has been related to altered cellular activation, we evaluated whether HP-BCD treatment could modulate monocyte response to inflammatory stimuli. Treatment of monocytes isolated from HIV-positive and HIV-negative donors with HP-BCD inhibited the expression of CD36 and TNF-α after LPS stimulation, independent of raft disruption. Accordingly, HP-BCD-treated cells showed significant reduction of TNF-α and IL-10 secretion, which was associated with lower mRNA expression. LPS-induced p38MAPK phosphorylation was dampened by HP-BCD treatment, indicating this pathway as a target for HP-BCD-mediated anti-inflammatory response. The expression of HLA-DR was also reduced in monocytes and dendritic cells treated with HP-BCD, which could hinder T cell activation by these cells. Our data suggest that, besides its well-known antiviral activity, HP-BCD could have an immunomodulatory effect, leading to decreased inflammatory responses mediated by antigen-presenting cells, which may impact HIV pathogenesis and AIDS progression.

**IMPORTANCE** Chronic immune activation is a hallmark of HIV infection and is often not controlled even in patients under antiretroviral therapy. Indeed, chronic diseases with inflammatory pathogenesis are being reported as major causes of death for HIV-infected persons. Hydroxypropyl-beta cyclodextrin (HP-BCD) is a cholesterol-sequestering drug that inhibits HIV replication and infectivity *in vitro* and *in vivo*. Recent studies have demonstrated the importance of cholesterol metabolism and content in different inflammatory conditions; therefore, we investigated the potential of HP-BCD as an immunomodulatory drug, regulating the activation of cells from HIV-infected patients. Treatment of monocytes with HP-BCD inhibited the expression and secretion of receptors and mediators that are usually enhanced in HIV patients. Furthermore, we investigated the molecular mechanisms associated with the immunomodulatory effect of HP-BCD. Our results indicate that, besides reducing viral replication, HP-BCD treatment may contribute to modulation of chronic immune activation associated with AIDS.

## Abstract (precis)

Chronic immune activation is a hallmark of HIV infection and is often not controlled even in patients under antiretroviral therapy. Indeed, chronic diseases with inflammatory pathogenesis are being reported as major causes of death for HIV-infected persons. Hydroxypropyl-beta cyclodextrin (HP-BCD) is a cholesterol-sequestering drug that inhibits HIV replication and infectivity *in vitro* and *in vivo*. Recent studies have demonstrated the importance of cholesterol metabolism and content in different inflammatory conditions; therefore, we investigated the potential of HP-BCD as an immunomodulatory drug, regulating the activation of cells from HIV-infected patients. Treatment of monocytes with HP-BCD inhibited the expression and secretion of receptors and mediators that are usually enhanced in HIV patients. Furthermore, we investigated the molecular mechanisms associated with the immunomodulatory effect of HP-BCD. Our results indicate that, besides reducing viral replication, HP-BCD treatment may contribute to modulation of chronic immune activation associated with AIDS.

## INTRODUCTION

HIV prevalence is rising in nearly every geographic region in the world due to the increased survival of patients treated with antiretroviral drugs (ARTs). The cause of death for most HIV-infected persons has changed from AIDS-related opportunistic infections to chronic diseases with inflammatory pathogenesis. Chronic immune activation is a hallmark of HIV infection, and increased frequency of activated immune cells is a strong predictor of disease progression (1–4). Importantly, ART treatment has a limited effect on immunological control, even when efficient viral suppression is achieved (5, 6).

The systemic inflammation observed in HIV-infected patients may result primarily from the activation of innate immune cells by the virus or by microbial products due to microbial translocation, impacting T cell activation (7–9). During HIV infection, a shift from classical monocytes (CD14^High^ CD16^−^) to inflammatory monocytes (CD14^+^ CD16^+^) and patrolling monocytes (CD14^High^ CD16^+^) is often observed (10). The latter cells express high levels of activation markers, such as CD80, CD86, and HLA-DR, and exhibit enhanced TNF-α production upon stimulation with bacterial lipopolysaccharides (LPS) (11). Increased levels of TNF-α in plasma are a major indicator of immune activation, and it was strongly correlated with activation and depletion of T cells in HIV-positive patients (12, 13). IL-10 levels, mainly produced by monocytes, are also augmented in HIV-infected patients and are correlated positively with viral load and negatively with CD4 T cell counts and function (14–16). Both IL-10 and TNF-α are biomarkers of early immune activation, and their levels are correlated with viral load and persistence (17).

Reciprocal regulation between immune and lipid metabolic function and content has been recently unraveling during monocyte/macrophage stimulation under different inflammatory conditions. Cholesterol crystals were associated with inflammasome activation in macrophages during atherogenesis (18), and oxidized cholesterol metabolites were strongly proinflammatory for different cell types, inducing the production of cytokines such as TNF-α and MIP-1β (19). Further, increased oxysterol levels, associated with repression of cholesterol synthesis, prevented inflammasome activation and restricted inflammation in experimental models of septic shock and autoimmunity (20, 21).

Monocytes from HIV+ patients express high levels of the lipid uptake receptor CD36, and it was inversely correlated with CD4^+^ T cell counts (22). Also, dysregulation of genes involved in lipid metabolism have been reported to be involved in the sustained immune activation and macrophage inflammatory phenotype in HIV patients (23). Antigen-presenting cells (APCs) derived from HIV-infected nonprogressors (NP) showed lower levels of membrane cholesterol than cells from progressors, and this phenotype was correlated with a decreased ability of NP APCs to mediate HIV trans infection of T cells (24, 25). These findings strengthened the fundamental role of cholesterol in viral replication and suggested that lower membrane cholesterol levels might contribute to delayed disease progression.

2-Hydroxypropyl-beta-cyclodextrin (HP-BCD) is a cholesterol-sequestering drug, which is being used in humans for several pharmaceutical applications and has also been tested in human clinical trials of Niemann Pieck C disease (NPCD), with a safe profile (26–28). Importantly, this agent has been extensively demonstrated to inactivate HIV *in vitro*, including ART-resistant virus (29–32). HP-BCD treatment also decreased virus infectivity and partially prevented mucosal transmission of SIV in a macaque model (32). Given that diminished cholesterol levels are associated with decreased virus replication and lower inflammatory response in different models, we hypothesized that treatment of HIV+ patients with HP-BCD could impair HIV disease progression by acting directly on virus particles and by downmodulating the inflammatory response.

Here, we investigated the impact of *in vitro* HP-BCD treatment on the activation profile of monocytes upon encounter with an inflammatory stimulus. Treatment of monocytes, obtained from HIV-positive and HIV-negative donors, with HP-BCD resulted in decreased expression of CD36 and remarkable inhibition of TNF-α and IL-10 secretion, independent of lipid raft disruption. The inhibition of TNF-α and IL-10 production was associated with decreased mRNA expression and downregulation of p38MAPK activation. The cells also showed decreased expression of HLA-DR upon HP-BCD treatment, suggesting that it could potentially affect T cell activation. Our data demonstrated that HP-BCD has an immunomodulatory effect, leading to a decreased inflammatory activation of antigen-presenting cells. Therefore, HP-BCD treatment may contribute to modulation of the chronic immune activation associated with AIDS.

## RESULTS

### HP-BCD decreased CD36 and TNF-α expression in monocytes obtained from chronic HIV patients.

**FIG 1:**
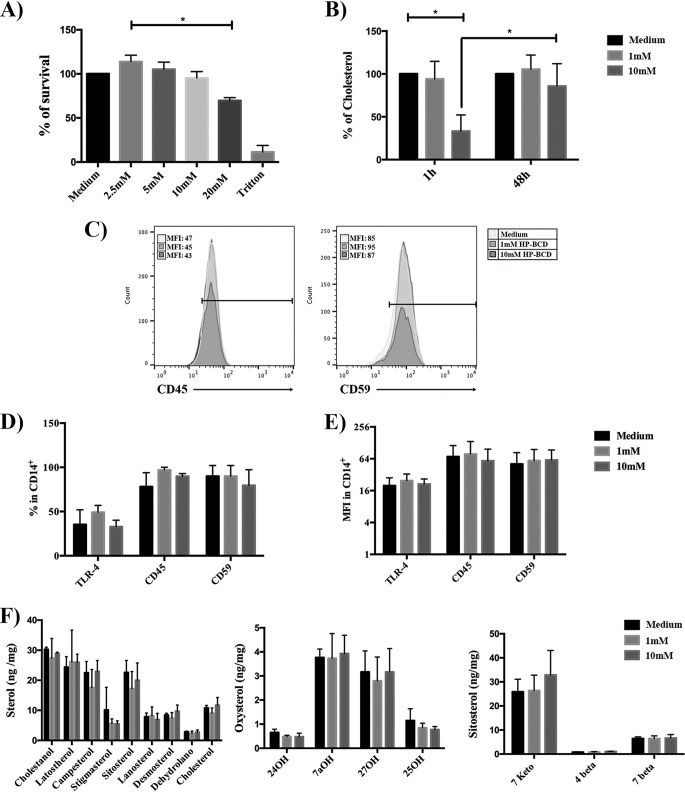
Analysis of cytotoxicity and kinetics of cholesterol depletion after HP-BCD treatment. (A) Monocytes were purified from HIV-negative donors and treated with the indicated concentrations of HP-BCD for 1 h. The cells were washed, the medium was substituted for HP-BCD-free complete medium, and the cells were cultured for an additional 48 h. As controls, cells were cultured with culture medium or incubated with Triton X-100. Cellular viability was analyzed using a Cell-Titer blue kit. (B) Monocytes were treated with 1 or 10 mM HP-BCD as in panel A. The amount of cholesterol was measured after 1-h treatment and after 48-h culture using the Amplex Red reagent. (C to E) Monocytes were treated with 1 or 10 mM HP-BCD as in panel A**.** (C) A representative histogram of CD14^+^ cells stained with CD59 and CD45 raft and nonraft markers is shown. (D and E) The frequency (as a percentage) of cells expressing CD45, CD59, and TLR-4 (D) and the level of expression (mean fluorescence intensity [MFI]) of each molecule (E) among CD14^+^-gated cells were analyzed by flow cytometry. (F) Monocytes were treated with 1 or 10 mM HP-BCD as in panel A**.** The amount of all sterols, oxysterols, and sitosterols were evaluated by GC-MS. The bars indicate the averages plus standard deviations (error bars) of the data obtained with cells from at least six individual donors. *, *P* values of <0.05.

To investigate whether HP-BCD would modulate the activation threshold of monocytes obtained from HIV patients, primary cells were cultured with different concentrations of HP-BCD for 1 h, the cells were washed, and the medium was substituted for complete medium with no HP-BCD. The cells were then cultured for an additional 48 h before stimulation with LPS, which was used as a surrogate for microbial activation. Initially, we established the maximum nontoxic concentration of HP-BCD and determined the kinetics of cholesterol recovery after cell treatment. HP-BCD was not toxic for primary monocytes even at 10 mM (Fig. 1A). Cell treatment with 10 mM HP-BCD induced 70% cholesterol reduction after 1-h treatment, with almost 100% recovery after 48 h of culture (Fig. 1B). The addition of 1 mM HP-BCD did not significantly deplete cholesterol at any time point analyzed (Fig. 1B). Cholesterol and raft recovery after 48-h culture was confirmed by staining the cells with anti-CD59 and anti-CD45 as raft and nonraft markers and with anti-TLR4. As demonstrated in Fig. 1C to E, no alteration in the frequency or expression level of either receptor was detected at the investigated time point. We also measured the concentrations of several sterol intermediates, oxysterols, and sitosterols in the cells at 48 h after HP-BCD treatment, and no significant differences for any of these lipids were observed (Fig. 1F).

**FIG 2:**
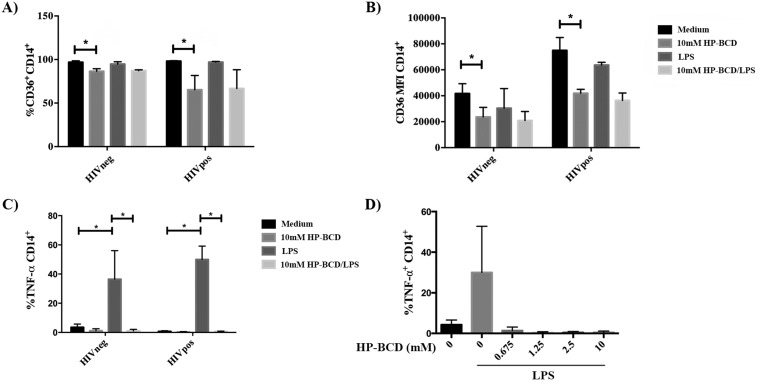
HP-BCD inhibited the expression of the activation markers CD36 and TNF-α in monocytes derived from HIV patients upon LPS stimulation. (A to C) Monocytes were purified from HIV-negative or HIV-positive donors and treated with 10 mM HP-BCD for 1 h. Cells were washed and then cultured in complete medium with no HP-BCD. After 48 h, the cells were stimulated with LPS for 8 h, and the expression of CD36 and TNF-α was evaluated by flow cytometry. (A and B) The frequency (as a percentage) of cells expressing CD36 (A) and the CD36 expression level (MFI) (B) among CD14^+^-gated cells. (C) Frequency of TNF-α-producing cells among CD14^+^-gated cells. (D) Cells obtained from HIV-positive patients were cultured with the indicated concentration of HP-BCD and stimulated with LPS as in panels A to C, and the frequency of TNF-α-producing cells among CD14^+^-gated cells was evaluated. The bars indicate the average and standard deviation of the data obtained with cells from at least six individual donors. *, *P* values of <0.05.

Monocytes were then pretreated or not with HP-BCD, and after culturing for 48 h in HP-BCD-free media, the cells were stimulated with LPS. Eight hours after LPS activation, the expression of CD36 and TNF-α were evaluated by flow cytometry. Pretreatment of the cells with HP-BCD decreased the frequency of CD36^+^ cells from almost 100% to 85% and 65% average in HIVneg and HIVpos donors, respectively. All the analyzed samples also demonstrated a reduced expression level of CD36 (mean fluorescence intensity) in both HIVneg and HIVpos donors, with an average reduction of 44% and 45%, respectively (Fig. 2A and B). Remarkably, HP-BCD treatment almost abolished intracellular TNF-α expression induced by LPS (Fig. 2C). Inhibition of TNF-α was detected even when HP-BCD was added at a concentration lower than 1 mM (Fig. 2D), supporting that this effect was not a simple consequence of membrane cholesterol depletion.

### HP-BCD inhibited the production of TNF-α and IL-10 at transcriptional levels.

**FIG 3:**
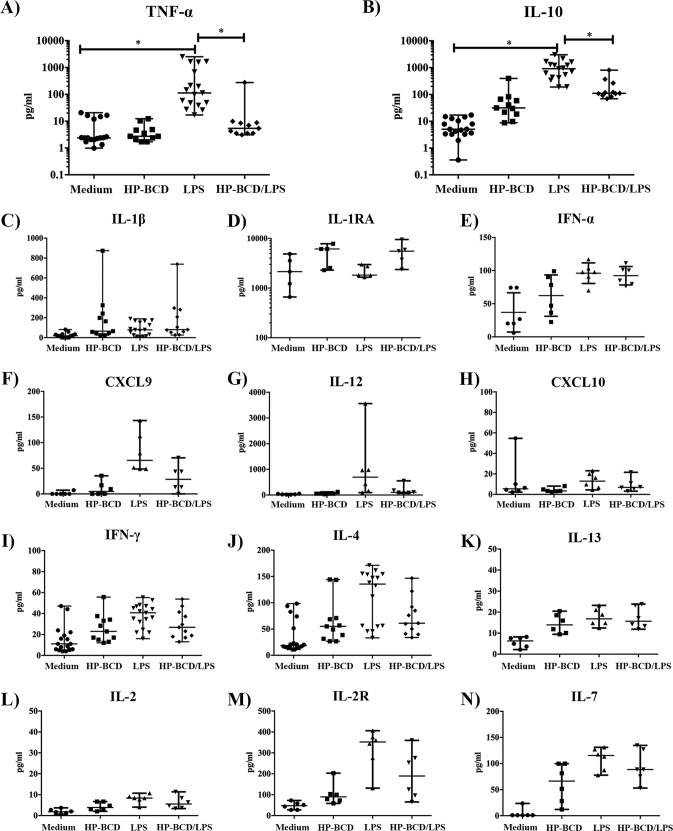
HP-BCD treatment downregulates the secretion of TNF-α and IL-10 in monocytes from HIV-positive donors. Monocytes were purified from HIV-positive donors and treated with 1 mM HP-BCD or not treated with HP-BCD for 1 h. The cells were washed and then cultured in complete medium for 48h. The cells were stimulated with LPS for 48 h, and the levels of TNF-α (A), IL-10 (B), IL-1β (C), IL-1RA (D), IFN-α (E), CXCL9 (F), IL-12 (G), CXCL10 (H), IFN-γ (I), IL-4 (J), IL-13 (K), IL-2 (L), IL-2R (M), and IL-7 (N) were measured in the supernatant cultures using Bioplex assay. The dots represent the data obtained from individual donors, and the average and standard deviation are indicated. *, *P* values of <0.05.

We then addressed whether HP-BCD would impact the secretion of TNF-α and other pro- and anti-inflammatory mediators induced by LPS. Monocytes obtained from HIV patients were pretreated or not with HP-BCD and stimulated with LPS. After 48 h, the secretion of multiple cytokines was measured by multiplex analyses of the supernatants (Fig. 3). HP-BCD treatment inhibited LPS-induced secretion of TNF-α and IL-10 in all donors analyzed, and the differences were statistically different (Fig. 3A and B). On the other hand, downregulation of IFN-γ was observed in only 1 donor out of 11 donors; IL-4 was downregulated in 2 out of 10 donors; an IL-2R was reduced in 2 out of 6 patients, whereas the secretion of IL-1β, IL-1RA, IL-2, IL-7, IL-12, IL-13, IP-10, MIG-1, and IFN-α were not affected in any of the donors analyzed (Fig. 3C to N). These data demonstrate that the drug specifically modulated TNF-α and IL-10 secretion but did not induce an overall monocytic anergic phenotype.

**FIG 4:**
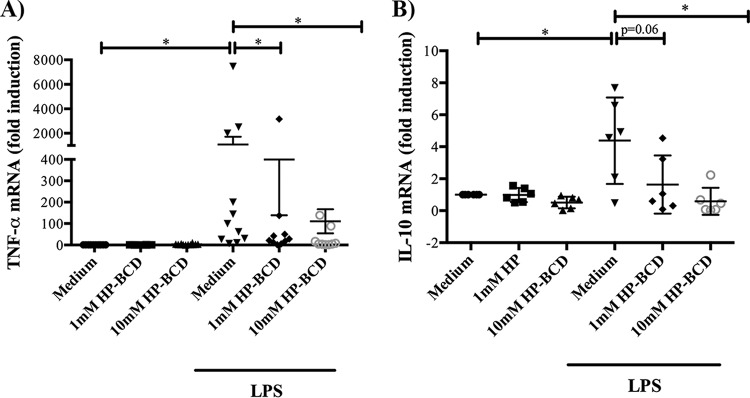
HP-BCD affects monocyte activation by downmodulating TNF-α and IL-10 mRNA expression. Monocytes were purified from HIV-negative donors and treated with 1 or 10 mM HP-BCD for 1 h. Cells were washed and then cultured in complete medium for 48 h. (A) Cells were stimulated with LPS for 4 h, and TNF-α mRNA levels were measured by RT-PCR. (B) Cells were stimulated with LPS for 24 h, and IL-10 mRNA levels were measured by RT-PCR. The dots represent the data obtained from individual donors, and the average and standard deviation are indicated. *, *P* values of <0.05.

The levels of expression of TNF-α and IL-10 were then accessed by measuring the respective cytokine mRNA levels after LPS stimulation of HP-BCD-pretreated cells. As expected, LPS induced the expression of TNF-α and IL-10 mRNAs, and both were greatly reduced when the cells were pretreated with HP-BCD (Fig. 4A and B), indicating that HP-BCD reduced LPS-mediated inflammatory response by decreasing transcription of the cytokine genes.

### HP-BCD treatment inhibits p38 MAPK activation induced by LPS.

**FIG 5:**
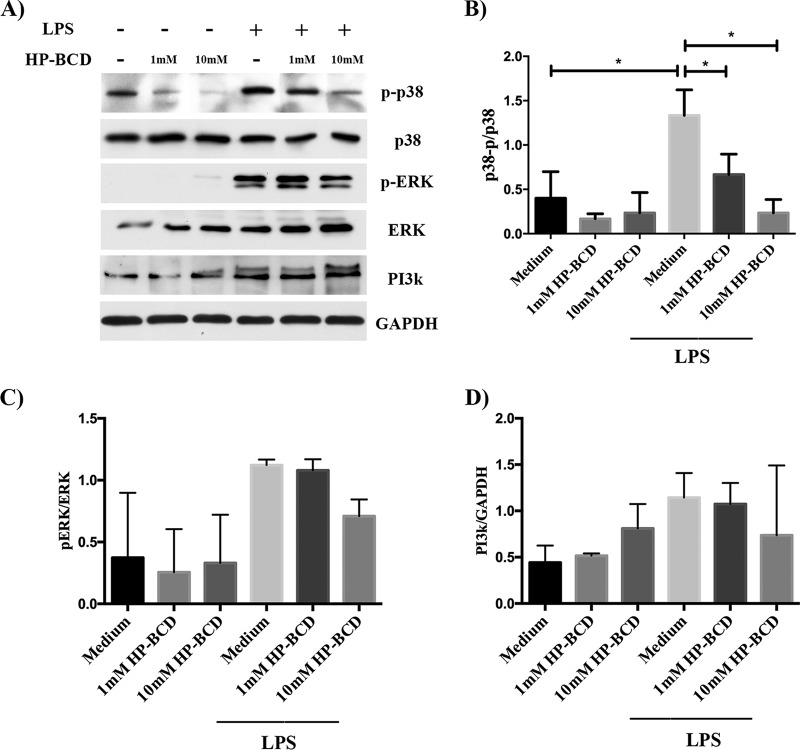
HP-BCD downregulates p38 phosphorylation induced by LPS, but not PI3K expression or ERK phosphorylation. Monocytes were purified from HIV-negative donors and treated with 1 or 10 mM HP-BCD for 1 h. Cells were washed, cultured in complete medium for 48 h, and stimulated with LPS for 30 min. The expression of p-p38, p38, pErk, Erk, PI3K, and GAPDH in the cell lysates was evaluated by Western blotting (WB), and the ratio of phosphorylated proteins in relation to nonphosphorylated or constitutive protein was determined using ImageJ software. (A) Representative WB raw data. (B and C) The ratio of phosphorylated p38 (p-p38) (B) and phosphorylated Erk (pErk) (C) in relation to unphosphorylated p38 and Erk, respectively, were calculated and represented. (D) The expression of PI3K in the cell lysates was normalized according to the expression of GAPDH. The bars indicate the averages and standard deviations of the data obtained with cells from four individual donors. *, *P* values of <0.05.

Monocyte stimulation by LPS-TLR4 engagement triggers distinct signaling pathways, resulting in increased expression of inflammatory and regulatory cytokines (33–35). Since PI3K activation is dependent on lipid raft recruitment (36), we initially investigated whether HP-BCD treatment would affect PI3K expression induced by LPS. Monocytes stimulated with LPS showed enhanced levels of PI3K, but it was not affected by HP-BCD (Fig. 5A and D). We then measured the levels of phosphorylated p38 (p-p38) and Erk (pErk), as an indication of MAPK activation. As expected, LPS stimulation induced increased expression of p-p38 and pErk. Activation of p38, but not Erk, was strongly inhibited in the cells previously treated with HP-BCD (Fig. 5A to C). These data suggest that HP-BCD treatment down-modulates specific signaling pathways, which in turn may explain the selective inhibition of TNF-α and IL-10, but not other cytokines.

### The expression of costimulatory molecules is impacted by HP-BCD treatment.

**FIG 6:**
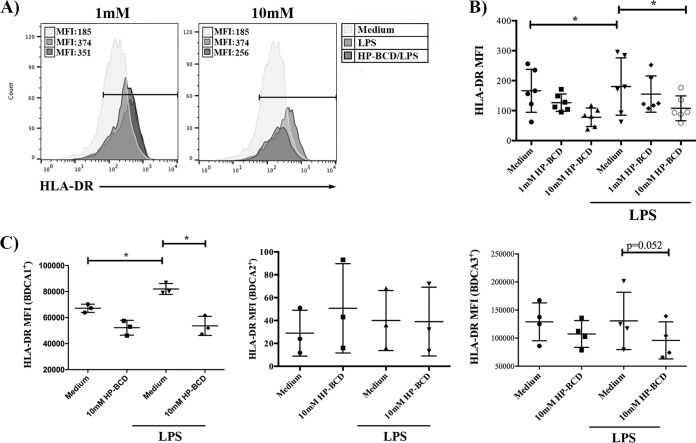
Treatment of monocytes with HP-BCD downregulates the expression of HLA-DR induced by LPS. CD14^+^ purified monocytes or residual CD14^−^ PBMCs, obtained from HIV-positive patients, were treated with HP-BCD for 1 h or not treated with HP-BCD. Cells were washed, cultured in complete medium for 48 h, and stimulated with LPS for an additional 24 h. Cultures of purified monocytes were stained with anti-CD14 and anti-HLA-DR; cultures of CD14^−^ PBMCs were stained with anti-BDCA1, anti-BDCA2, or anti-BDCA3 and anti-HLA-DR. The expression of HLA-DR among each cell subpopulation was evaluated by flow cytometry. (A) Representative histogram overlays indicating the mean fluorescence intensity (MFI) of HLA-DR expression among CD14^+^ cells. (B) MFI of HLA-DR in CD14^+^-gated population by individual donor samples. (C) MFI of HLA-DR in CD14^−^ BDCA1, BDCA2, or BDCA3^+^ cells by individual donor samples. *, *P* values of <0.05.

Since chronic activated monocytes also impact T cell activation by direct stimulation through immunological synapses, we addressed whether HP-BCD affected the costimulatory phenotype of antigen-presenting cells. Flow cytometry analysis demonstrated that HP-BCD-treated monocytes showed decreased expression of HLA-DR upon LPS stimulation (Fig. 6A and B). We also investigated whether other myeloid cells would be affected by evaluating the expression of HLA-DR among BDCA1-, BDCA2-, and BDCA3-expressing CD14-negative cells. Stimulation with LPS slightly enhanced HLA-DR expression in BDCA1+ myeloid DCs, and HP-BCD pretreatment abolished this effect (Fig. 6C). These findings suggest that HP-BCD may impair the ability of LPS-stimulated myeloid cells to activate T cells, which could further contribute to downregulate chronic immune activation.

## DISCUSSION

This study demonstrated that treatment of monocytes from HIV-infected patients with the cholesterol-sequestering drug HP-BCD inhibits their stimulation by LPS, resulting in decreased secretion of TNF-α and IL-10. Membrane cholesterol and lipid raft organization are cellular elements essential for replication of many enveloped viruses, and HP-BCD has been demonstrated to inhibit HIV infection and to inactivate free HIV particles in *in vitro* and *in vivo* models (29–32). In addition, HIV infection is associated with higher atherogenic risk and cardiovascular disease, which might be related to altered uptake, synthesis, or efflux of cholesterol, evidenced by accumulation of foam cells (37, 38). Recent evidences have demonstrated that cellular lipid metabolism, specifically metabolism of cholesterol and other sterols impacts distinct signal transduction pathways during the activation of immune cells (18–20). These findings motivated us to investigate the effect of HP-BCD, in an *in vitro* model, as a potential anti-HIV drug able to act on the chronic immune activation experienced by HIV-infected patients.

Microbial translocation and circulating LPS are markers of HIV progression and are associated with the production of inflammatory mediators by monocytes, activation of T cells, and exacerbation of inflammation (39, 40). LPS sensing through TLR4 induces the secretion of several inflammatory cytokines, including TNF-α, which is one of the main mediators detected in plasma samples from chronic HIV patients. Activated monocytes were also associated with increased plasma IL-10 levels in HIV patients, which were correlated with CD4 T cell dysfunction and disease progression (14–16).

The importance of inflammatory mediators produced by monocytes/macrophages for the establishment of chronic immune activation during HIV progression was further evidenced in nonpathogenic SIV infection. Monocytes obtained from SIVsm-infected macaques showed lower TNF-α secretion in response to LPS compared to monocytes obtained from HIV-infected humans or SIVmac-infected rhesus macaques. Neutralization of TNF-α resulted in decreased activation of CD8^+^ T cells, indicating that monocyte activation was associated with enhanced stimulation of T cells (41).

The treatment of monocytes with HP-BCD *in vitro* downregulated TNF-α and IL-10 production independently of raft disruption. These findings are in accordance with a previous report of an experimental model of LPS-induced fatal shock in mice, in which mice treated with cyclodextrin exhibited reduced blood TNF-α levels and mortality (42). Also, the addition of cyclodextrin to murine macrophage cell lines did not affect localization or expression of CD14 in lipid rafts or the membrane levels of TLR4-MD2 complexes but inhibited LPS-induced TNF-α secretion (42).

Recent evidences demonstrated that PRR immune signaling may cross talk with other metabolic pathways. The lipid uptake receptor CD36 was previously associated with upregulation of TLR2/4 and increased LPS responsiveness (43), and accumulation of saturated fatty acids enhanced TNF-α secretion (44). Consistent with previous reports, the cells obtained from HIV patients in this study presented increased basal expression of CD36 (22, 23) (Fig. 2B), and HP-BCD strongly inhibited CD36 expression, what may contribute to decreased TNF-α secretion. Interestingly, CD36 may also be upregulated by TNF-α (45). Therefore, decreased TNF-α production mediated by HP-BCD might explain lower CD36 expression. Importantly, decreased CD36 expression might be expected to modulate the dyslipidemia and attenuate the atherogenic risk in HIV patients. Hence, HP-BCD treatment could potentially modulate atherogenic and inflammatory pathways through its effect on cellular signaling pathways.

The impaired ability of HP-BCD-treated monocytes to respond to LPS resulted from transcriptional regulation of IL-10 and TNF-α. LPS-induced upregulation of TNF-α and IL-10 mRNA in PBMCs was shown to be dependent on activation of different MAP kinases (34, 35). MAPKs were also involved in CD36-mediated cell signaling (43). It was previously reported that monocytes cultured with high-density lipoprotein (HDL), which mediates cholesterol efflux, inhibited LPS- or IFN-γ-driven M1 polarization and decreased expression of TNF-α, IL-6, and CCL2 genes (46). The HDL-mediated effect was shown to be dependent on inhibition of caveolin expression and Erk1/2 phosphorylation; however, unlike our model, HDL was added to the cultures simultaneously with LPS. We did not detect any alteration in Erk phosphorylation level, but HP-BCD strongly inhibited the phosphorylation of p38 induced by LPS, indicating that p38, but not all MAPK, were regulated by HP-BCD, and further demonstrating that cholesterol perturbation affects macrophage differentiation.

Despite evidence indicating that the PI3K/akt pathway is a major regulator of IL-10 production, we did not detect any significant alteration in PI3K expression in our model, indicating that other mechanisms might be involved in the regulation of LPS stimulation by HP-BCD. Secreted IL-10 may impair its own production in a negative-feedback loop by downregulating p38 phosphorylation (33). It is possible that a modest IL-10 secretion induced by HP-BCD downmodulated other signal transduction pathways controlling the production of TNF-α and IL-10 itself. Further analysis of cell signaling pathways induced by HP-BCD will be required to dissect the mechanisms by which the drug influences cell activation and inflammation.

Finally, we demonstrated that HP-BCD treatment affected the expression of HLA-DR in monocytes and myeloid DCs activated by LPS. HIV patients often present a lower frequency of certain DC subpopulations, but circulating DCs are able to vigorously respond to stimulation, what may impact chronic immune activation (47, 48). HP-BCD-mediated downregulation of HLA-DR could then contribute to the control of nonspecific T cell activation.

Previous studies demonstrated that BCD showed a direct effect on HIV replication by different mechanisms of action, including: (i) free virus inactivation (29, 30); (ii) inhibition of HIV entry/fusion (29, 49, 50); (iii) inhibition of virus release (30). Similar to the experiments performed here, all these studies used BCD at concentrations up to 20 mM (typically 10 mM), which showed no toxicity to different cell types analyzed, including primary macrophages or PBL (49, 50). A 20 mM dose of HP-BCD (or higher) was also tested in experimental mouse and macaque models, where topical application of the agent was demonstrated to prevent HIV and SIV transmission with no toxicity to the tissue (32, 51). Importantly, HP-BCD has been extensively reported to be safe for human use as an oral, topical, and parenteral agent (26–29). In addition, a phase 1/2a clinical trial designed to test the safety and effectiveness of intrathecal injections of HP-BCD for the treatment of NPCD patients was recently successfully completed (27, 28). Therefore, the data presented in this study provide substantial support for the evaluation of HP-BCD as an antiretroviral drug potentially able to reduce viral replication (29–32) and control HIV-associated chronic immune activation that aggravates HIV comorbidities and AIDS progression.

## MATERIALS AND METHODS

### Ethical statement and sample description.

Blood samples from deidentified HIV-negative donors were obtained from the Hemotherapy Service at the Hospital Universitário Clementino Fraga Filho (HUCFF) of Universidade Federal do Rio de Janeiro (UFRJ). The study protocol was approved by the Experimental Ethics Committee of UFRJ (permit 105/07). Blood samples from HIV-infected subjects were obtained from University of California Davis Medical Center and included patients who were on antiretroviral therapy and had undetectable HIV RNA (Table 1). The study protocol was approved by institutional review board at the University of California (protocol 219307).

**TABLE 1:** Description of the patients enrolled in the study

Patient	Age (yr)	Gender	HIV viral load (copies/ml)	CD4 count (mm^3^)	Yr of diagnosis	ART regimen
1	51	M	Undetectable	1,214	2008	Epzicom, atazanavir/ritonavir
2	59	M	Undetectable	766	2006	Darunavir, dolutegravir, truvada
4	46	M	Undetectable	153	2011	Elvitegravir, tonofovir, emtricitabine, ALA
5	63	M	<20	790	2008	Efavirenz, emtricitabine, tenofovir
6	54	M	<20	698	2008	Abacavir, dolutegravir, lamivudine
8	58	M	<20	916	2014	Abacavir, lamivudine
9	66	M	<20	417	2011	Efavirenz, emtricitabine, tenofovir
10	63	M	<20	445	2008	Truvada, emtricitabine, tenofovir
11	60	M	64	184	2011	Truvada, emtricitabine, tenofovir
12	51	M	<20	441	2005	Nevirapine, lamivudine, and azidovudin
13	66	M	68	288	1989	Abacavir, dolutegravir, lamivudine
14	46	M	<20	514	2015	Emtricitabine, rilpivirine, tenofovir, ALA
15	52	M	<20	1,191	2008	Ritonavir, atazanavir
16	52	M	1927	148	2008	Ritonavir, darunavir
17	62	M	<20	430	1999	Truvada, maraviroc
19	45	M	Undetectable	880	1994	Atazanavir, ritonavir
20	44	M	<20	929	2012	Emtricitabine, rilpivirine, tenofovir, ALA
21	47	M	93	1,645	1999	Lopinavir, ritonavir
22	49	F	<20	904	2006	Abacabir, dolutegravir

### Monocyte and dendritic cell isolation and HP-BCD treatment.

Fresh peripheral blood mononuclear cells (PBMCs) were obtained by Ficoll-Hypaque density gradient centrifugation. Monocytes were negatively isolated from PBMCs with CD14 enrichment kits (StemCell Technologies, Vancouver, Canada), according to the manufacturer’s protocol. The cells were cultured with RPMI supplemented with L-glutamine and 10% FCS (complete medium). Dendritic cells were obtained as residual cells from monocyte purifications and cultured with complete medium. Evaluation of DC subpopulations were performed by selectively analyzing BDCA-1^+^, BDCA-2^+^, or BDCA-3^+^ cells as described below.

The cells were treated with the indicated concentrations of 2-hydroxypropyl-beta-cyclodextrin (HP-BCD) (Cyclodextrin Technologies Development, Inc. [CTD], High Springs, FL) for 1 h in serum-free medium. The cells were washed and then cultured in complete medium with no HP-BCD for an additional 48 h. Forty-eight hours after treatment, the cells were stimulated with 20 ng/ml of LPS for different time periods, according to the analysis to be performed.

### Cell viability.

Monocytes were treated with different concentrations of HP-BCD as described, and cell viability was assessed after 48 h of culture using a Cell-Titer blue kit (Promega, Madison, USA), according to the manufacturer’s protocol.

### Cholesterol measurement.

Monocytes were treated with different concentrations of HP-BCD for 1 h in serum-free medium. The cells were washed and then cultured in complete medium for 48 h. Cholesterol content was measured 1 h after treatment and after 48 h of culture using Amplex Red reagent (Thermo Fisher Scientific Inc., Pittsburgh, PA, USA), according to the manufacturer’s protocol.

The analysis of other sterols was performed on the cell pellets dried in a Savant SpeedVac concentrator (Thermo Fisher Scientific Inc.) for 24 h. Cholesterol, noncholesterol sterols, and oxysterols were extracted from dry aliquots (dry weight) using Folch reagent (chloroform-methanol [2:1 {vol/vol}] with 0.25 mg BHT added per ml solvent) per 10 mg dried cell pellets. Extraction was performed for 12 h at 4°C in a dark cold room. One milliliter of the Folch reagent underwent alkaline hydrolysis, extraction of the free sterols and oxysterols, silylation to their corresponding (di)trimethylsilyl ethers prior to gas chromatographic separation and detection by mass selective detection (for noncholesterol sterols or oxysterols using epicoprostanol and the corresponding deuterium-labeled oxysterols as internal standards, respectively) as described in detail previously (52).

### Expression of surface proteins and analysis of cell activation by flow cytometry.

Monocytes were treated with HP-BCD as described. To analyze the expression of CD36 and TNF-α, the cells were stimulated with LPS for an additional 8 h, and 10 μg/ml of Brefeldin A was present for the last 4 h. To analyze the expression of costimulatory molecules, cells were stimulated with LPS for 24 h. The cells were incubated with APC-, FITC-, PE-PerCP-, PECy7-, and APC-Cy7-conjugated antibodies against CD14, CD36, CD59, CD45, BDCA1, BDCA2, BDCA3, HLA-DR, and CD86 (eBioscience, California, USA) for 1 h at 4°C. For intracellular TNF-α staining, cells were permeabilized and incubated with PerCP-conjugated anti-TNF-α (eBioscience) for 1 h at 4°C. Event acquisition and analyses were performed with an Attune flow cytometer (Thermo Fisher Scientific Inc.) and FlowJo software (Tree Star, Inc., Oregon, USA).

### Cytokine secretion.

Monocytes were treated with HP-BCD as described. After 48 h, cells were stimulated with LPS for an additional 48 h. Culture supernatants were harvested, and the secretion of IL-1β, IL-1RA, IL-2, IL-2R, IL-4, IL-7, IL-10, IL-12, IL-13, IP-10, MIG-1, TNF-α, IFN-α, and IFN-γ was measured by Bioplex assay (Bio-Rad, California, USA), according to the manufacturer’s protocol.

### Expression of TNF-α and IL-10 mRNA.

Monocytes were treated with HP-BCD as described. Then, the cells were stimulated with LPS for 4 h or 24 h before analyzing the expression of TNF-α or IL-10 mRNAs, respectively. RNA was isolated from the cell lysates using TRIzol reagent (Thermo Fisher Scientific Inc.), according to the manufacturer’s instructions. First-strand cDNA was synthesized using a High-Capacity cDNA Archive kit (Thermo Fisher Scientific Inc.), according to the manufacturer’s instructions. The cDNA was subjected to real-time PCR using Power SYBR Green PCR master mix reagent (Thermo Fisher Scientific Inc.) using the following primers: TNF-α sense (5′-CAG AGG GAA GAG TTC CCC AGG GAC-3′), TNF-α antisense (5′-CCT TGG TCT GGT AGG AGA CGG C0, IL-10 sense (5′- AAT AAG GTT TCT CAA GGG GCT-3′), IL-10 antisense (5′-AGA ACC AAG ACC CAG ACA TCA A-3′), GAPDH sense (5′-GTG GAC CTG ACC TGC CGT CT-3′), and GAPDH antisense (5′-GGA GGA GTG GGT GTC GCT GT-3′). The reaction was carried out in a StepOnePlus real-time PCR system (Thermo Fisher Scientific Inc.), and the samples were subjected to 50°C for 2 min, 95°C for 10 min, and 40 cycles, with 1 cycle consisting of denaturation (95°C, 15 s), primer annealing (55°C, 30 s), and primer extension (60°C, 1 min). The samples were subjected to a melting curve analysis to eliminate primer dimers: 95°C for 15 s, 60°C for 1 min, and 95°C for 15 s. The comparative *C_T_* method (ΔΔCt) was used to quantify gene expression levels with GAPDH used for normalization.

### Analysis of PI3K and MAPK expression and phosphorylation by Western blotting.

Monocytes were treated with the indicated concentrations of HP-BCD as described. Then the cells were stimulated with LPS for 30 min. Cellular extracts were subjected to SDS-PAGE, followed by electrotransfer to a nitrocellulose membrane (Thermo Fisher Scientific Inc.). The membranes were incubated with anti-PI3K, anti-p-p38, anti-p38, anti-pERK, anti-ERK, and anti-GAPDH antibodies (Cell Signaling Technologies, Boston, MA, USA), followed by incubations with HRP-conjugated secondary antibodies (Santa Cruz Biotechnology). Super Signal West Pico chemiluminescent substrate (Thermo Fisher Scientific Inc.) was used for protein detection according to the manufacturer’s instructions. The ratio of phosphorylated proteins in relation to nonphosphorylated or constitutive protein was determined using ImageJ software.

### Statistical analysis.

Data were analyzed using the GraphPad Prism software (GraphPad Software, San Diego, CA, USA). Comparisons among groups were performed by two-way ANOVA; *P* values of <0.05 were considered statistically significant.
